# Prevalence of Traumatic Experiences in South Korean Adults

**DOI:** 10.3390/ijerph17113959

**Published:** 2020-06-03

**Authors:** Sunah Kim, Go-Un Kim, Suin Park

**Affiliations:** 1 College of Nursing, Yonsei University, Seoul 03722, Korea; PSY0962@yuhs.ac (S.K.); kgudfc@naver.com (G.-U.K.); 2 Mo-Im Kim Nursing Research Institute, College of Nursing, Yonsei University, Seoul 03722, Korea; 3 College of Nursing, Kosin University, Busan 49267, Korea

**Keywords:** psychological trauma, epidemiology, age groups, gender

## Abstract

Although a variety of traumatic experiences can impact health over the lifetime, little is known about the prevalence of such experiences in South Korea. The purpose of this study was to examine the frequencies of traumatic experiences and their differences by gender and age. South Korean adults (*N* = 3000) aged 19–65 years completed a self-report survey assessing childhood and adulthood trauma and sociodemographic characteristics. Overall, 79.5% of the participants reported at least one traumatic experience in adulthood. Unemployment or job loss and academic or work difficulties were commonly reported. The most distressing adulthood trauma was the unexpected death of a loved one, followed by diseases in loved ones. Childhood trauma was higher in males than in females. In males, childhood trauma was higher at a younger age, but in females, it was higher at an older age. Adulthood trauma was higher in females than in males and at older ages for both males and females. The current findings demonstrate the differences in traumatic experiences by gender and age in the South Korean general population. These results could help improve assessment of and targeted intervention for psychological trauma through trauma-informed strategies in public health practice.

## 1. Introduction

Traumatic experiences are a highly prevalent global phenomenon [1]. In the World Mental Health Survey, 70.4% of the participants reported experiencing at least one lifetime traumatic event, with exposure rates ranging from 28.6% in Bulgaria to 84.6% in Ukraine [1]. Further, the cross-national lifetime prevalence of posttraumatic stress disorder (PTSD) was 3.9% overall and 5.6% among respondents exposed to trauma [2]. Trauma is the result of a harmful or life-threatening event that persistently affects an individual’s functioning and holistic well-being [3]. Traumatic events appear in a variety of forms, including witnessing death or serious injury, the unexpected death of a loved one, threatening situations associated with physical violence, sexual violence, verbal abuse, or bullying within the workplace [1,4,5].

Trauma-related distress substantially affects victims’ health, and delayed treatment and care can lead to chronic physical and mental health problems [6,7,8,9]. Childhood trauma affects normal development and growth in a wide range of areas such as delayed cognitive development and psychological distress, and it adversely affects long-term individual outcomes [9]. Childhood maltreatment has been reported to have a negative impact on mental health, including depression and anxiety [7,8]. Indeed, childhood abuse has been reported to be associated with increased physical health problems in adulthood [6,10]. In terms of adulthood trauma experiences, increased physical and mental health symptoms have been reported in people who have experienced spousal violence, bullying in the workplace [5,11,12], and job-related traumatic events, such as firefighters [13]. In addition, stressful life events in adulthood have been associated with the onset of chronic fatigue syndrome [14].

Trauma-informed care (TIC) is a framework that delivers services to individuals who have experienced trauma and guides organizations within various healthcare settings [3,15]. The Substance Abuse and Mental Health Services Administration (SAMHSA) [3] reported that TIC involves four assumptions: to realize trauma and its influence on people; to recognize signs of trauma such as gender, age, or other specific factors; to respond to traumatized people by applying integrated knowledge about trauma; and to resist re-traumatization of both individual and staff. A better understanding of traumatic experiences can lead to TIC and better treatments in the public health system [16,17]. Thus, it is necessary to acquire the relevant information to develop and provide preventive health services for trauma exposure [15,16,17]. 

Although traumatic experiences are known to predict an increased risk of poor health and psychological distress, knowledge related to the various characteristics of traumatic experiences is limited among the general South Korean population. Previous studies in South Korea reported the adverse effects of traumatic experiences such as intimate partner violence and work-related trauma on mental health [12,13]. Other studies examined the frequencies of different traumatic experiences or their impact on mental health [18,19]. However, these studies were conducted with relatively smaller samples or limited to early adulthood traumatic experiences. Moreover, they did not examine differences by gender and age. Based on these previous studies, we targeted large-scale samples to investigate the prevalence with specific and systematic information regarding frequency of traumatic experiences and their differences by sociodemographic characteristics. 

Therefore, the aim of the current study was to describe the frequency of traumatic experiences and their differences with respect to gender and age using a large community sample in South Korea. Our research would contribute to promote trauma-informed care and better treatment in the public health system in South Korea.

## 2. Materials and Methods 

### 2.1. Study Design and Participants

A cross-sectional research design was used to study 3000 participants in South Korea (male = 1500; female = 1500). We used quota sampling stratified by gender and age. The genders were divided into four age groups (19–29, 30–39, 40–49, and 50–65 years), with each group representing 25% of the sample. The inclusion criteria were as follows: (1) aged 19–65 years, (2) capable of understanding the survey questions, and (3) able to use the internet and e-mail. Those who did not agree to participate in the study were excluded.

### 2.2. Instruments

#### 2.2.1. Traumatic Experiences

We focused on traumatic experiences in childhood as well as in adulthood. First, childhood trauma was assessed using the Korean version of the Childhood Trauma Questionnaire-Short Form (CTQ-SF) [20], originally developed by Bernstein et al. [21]. The CTQ-SF consists of 28 items designed to measure five categories of childhood abuse and neglect by a family member: physical abuse, emotional abuse, sexual abuse, physical neglect, and emotional neglect. Each of these categories consists of five items. Three additional minimization/denial validity items for detecting underreporting of abuse are included [22]. Items are rated on a five-point Likert scale (1 = never true; 5 = very often true), and the total score was obtained by summing up each item’s score. The total score ranged from 25 to 125 with higher scores indicating higher levels of severity. The overall Cronbach’s alpha coefficient for the Korean CTQ-SF in the present study was 0.94 (individual subscales: physical abuse = 0.89, emotional abuse = 0.86, sexual abuse = 0.91, physical neglect = 0.70, emotional neglect = 0.89). 

Second, to measure adulthood trauma, we revised the Traumatic Events List Questionnaire first developed by Song [23] and later revised by Shin [24]. The Adulthood Trauma Questionnaire (ATQ) used in this study excluded three items—“Accidents and injuries”, “Natural disaster”, and “Childhood maltreatment”—from the Traumatic Events List Questionnaire. This is because the first two were considered as a disaster, while we studied the third in detail using the CTQ-SF. We added three items on “Verbal threats or violence”, “Physical threats or violence”, and “Witness or awareness of suicide of other persons” and one item on other traumatic events. Two items were added to explore the utilization of mental health services associated with adult trauma: “After any of the traumatic experiences mentioned previously, did you use mental health services?” and “If you did not use mental health services, what was the reason?” The ATQ consists of 16 items measuring the experience and degree of traumatic distress on each item. For each item, 0 represented no experience of trauma. If there was a traumatic experience, the degree of distress was rated on a five-point Likert scale (1 = very little distress; 5 = extreme distress) for each item. The total score of adulthood trauma was obtained by summing up the distress scores of each item. The total scores ranged from 0 to 80 with higher scores indicating greater severity. The Cronbach’s alpha coefficient for the ATQ was 0.78 in the current study.

#### 2.2.2. Sociodemographic Characteristics

We collected the following variables as sociodemographic characteristics: gender, age, living status, education, employment status, and economic status. 

### 2.3. Ethical Considerations

The Institutional Review Board of the Yonsei University Health System in South Korea (Y-2018-0063) approved this study. Prior to participating in the survey, respondents were given an explanation of the purpose and procedure of the study. Further, they were assured of anonymity, confidentiality, and voluntary participation and that they could withdraw from the survey at any time without any penalty. 

### 2.4. Data Collection

The data were collected in July 2018. All participants were recruited through an online polling agency, “Market Link” (www.marketlink.co.kr), which hosted the survey panel to sample from a pool of over 500,000 people. After consultation with the administrator of the agency, the notice of participation in the research was announced to the panels via email through this site, and those who wished to participate in the research voluntarily completed the online questionnaire via the link provided in the notice. If there were any enquiries about the research description, informed consent, or questionnaire, the researcher’s phone number and e-mail were provided. Once the participant provided written informed consent, he/she could begin working on the questionnaire. The online questionnaire could be accessed from a computer or mobile phone in an environment where Internet access was available. The online surveys were designed to disallow participants from moving on to the next page without answering all the questions on the current page, thus ensuring higher completion rates. Duplicate accounts were eliminated using real name authentication, mobile phone authentication, and Internet Protocol address according to the survey platform policy. 

### 2.5. Data Analysis

All data were analyzed using the IBM SPSS Statistics version 24 (IBM Inc., Armonk, NY, USA). Descriptive statistics were performed on sociodemographic characteristics as well as on trauma-related variables. Kolmogorov–Smirnov tests were used to assess the normality of the quantitative data distribution [25]. Data with normal distribution were presented as mean and standard deviations (SDs). Data with abnormal distribution were presented as medians and interquartile ranges (IQRs). Since trauma-related variables were abnormally distributed, we used non-parametric tests. Mann–Whitney U tests and Kruskal–Wallis tes were performed to identify differences in trauma-related variables by sociodemographic characteristics. Mann–Whitney U tests and *χ*^2^ tests were used to identify differences in trauma by gender. Mann–Whitney U tests and Spearman’s rank correlation were used to examine differences in trauma by gender and age. A *p* < 0.05 was considered statistically significant.

## 3. Results

### 3.1. Sociodemographic Characteristics and Differences in Trauma-Related Variables by Sociodemographic Characteristics

The mean age of the 3000 participants was 40.03 years (*SD* = 11.54 years; range = 19–65). As shown in Table 1, over half of the respondents (53.2%; *n* = 1596) lived with a spouse; 82.8% (*n* = 2485) had higher education, and 73.5% (*n* = 2206) were employed. More than one-third of the participants reported high economic status (38.3%). Regarding trauma-related variables, the median score of childhood trauma was 42 (*IQR* = 28) and the median score of adulthood trauma was 11 (*IQR* = 17). 

Childhood trauma scores were significantly higher in males than in females (*U* = 1,054,371.50, *p* = 0.003), while adulthood trauma distress scores were higher in females than in males (*U* = 993,636.00, *p* < 0.001). Childhood and adulthood trauma scores were found to be significantly different according to the age groups (*χ*^2^*_kw_*(3) = 9.37, *p* = 0.025; *χ*^2^*_kw_*(3) = 20.18, *p* < 0.001, respectively). Both childhood and adulthood trauma scores were significantly higher in participants who lived without a spouse than in those living with a spouse (*U* = 1,044,914.50, *p* = 0.001; *U* = 1,022,598.00, *p* < 0.001, respectively), and in those who graduated high school or below than college or above (*U* = 551,847.00, *p* < 0.001; *U* = 579,992.50, *p* = 0.001, respectively). Those with low economic status had the highest scores in childhood and adulthood trauma (*χ*^2^*_kw_*(2) = 63.88, *p* < 0.001; *χ*^2^*_kw_*(2) = 19.49, *p* < 0.001, respectively). There were no differences in the trauma scores by employment status. 

### 3.2. Characteristics of Childhood and Adulthood Trauma and Its Differences by Gender

Table 2 depicts the characteristics of childhood and adulthood trauma and its differences by gender. Regarding the subscales of CTQ, males scored significantly higher than females on physical abuse (*U* = 1,005,371.50, *p* < 0.001), sexual abuse (*U* = 1,031,776.00, *p* < 0.001), and physical neglect (*U* = 946,396.50, *p* < 0.001). Emotional abuse and neglect did not differ significantly between males and females. 

Of all participants, 79.5% (*n* = 2386) experienced at least one among the 16 ATQ items, with females reporting more traumatic experiences than males (*χ*^2^(1) = 13.10, *p* < 0.001). The most commonly experienced ATQ items were unemployment or job loss (*n* = 1240, 41.3%), academic or work difficulties (*n* = 1168, 38.9%), and breakdown of interpersonal relationships (*n* = 1146, 38.2%). The most distressing traumatic experiences reported were the unexpected death of a loved one (mean = 4.54, *SD* = 0.76) followed by disease in a loved one (mean = 4.32, *SD* = 0.82) and financial difficulties (mean = 4.30, *SD* = 0.89). Females reported more experiences of sexual harassment or sexual abuse (*χ*^2^(1) = 102.67, *p* < 0.001), breakdown of interpersonal relationships (*χ*^2^(1) = 20.33, *p* < 0.001), bullying in school or at the workplace (*χ*^2^(1) = 22.96, *p* < 0.001), betrayal (*χ*^2^(1) = 7.60, *p* = 0.006), divorce or separation (*χ*^2^(1) = 22.80, *p* < 0.001), academic or work difficulties (*χ*^2^(1) = 7.26, *p* = 0.007), and witnessing or being aware of someone committing suicide (*χ*^2^(1) = 11.30, *p* = 0.001; Table 2). 

Of the participants who experienced at least one adulthood trauma, only 7.1% (*n* = 170) reported receiving mental health services after the experience. Among respondents who did not use mental health services after adulthood trauma, 64.3% (*n* = 1425) believed that their condition would improve over time, followed by 10.8% (*n* = 240) reporting time or financial constraints and 9.5% (*n* = 210) citing lack of information about services (Table 2).

### 3.3. Differences in Childhood and Adulthood Trauma by Gender and Age

Table 3 presents differences in childhood and adulthood trauma scores according to gender and age. Childhood trauma scores were higher in males than in females in the 19–29 and 30–39 age groups (*U* = 62,298.50, *p* = 0.007; *U* = 61,121.50, *p* = 0.002, respectively). In males, childhood trauma scores were generally higher in younger participants (*r* = −0.07; *p* = 0.008). In females, however, childhood trauma scores were generally higher in older participants (*r* = 0.05; *p* = 0.039). Adulthood trauma scores were significantly higher in females than in males in the 19–29, 30–39 and 50–65 age groups (*U* = 58,032.00, *p* < 0.001; *U* = 59,850.50, *p* < 0.001; *U* = 62,824.00, *p* = 0.011, respectively). Adulthood trauma scores were generally higher in older participants in both males (*r* = 0.11, *p* < 0.001) and females (*r* = 0.05, *p* = 0.036).

## 4. Discussion

This study examined the frequencies of traumatic experiences and their differences by gender and age using a large community sample in South Korea. In this study, the prevalence of exposure to at least one traumatic experience in adulthood was 79.5%. This result was higher than those reported by the World Mental Health Survey [1] and Netherlands Mental Health Survey and Incidence Study-2 [26]. The current result was also higher than the prevalence reported in a previous study in South Koreans [18]. The most frequently experienced events in the present study were unemployment or job loss, followed by academic or work difficulties. This result is inconsistent with a previous study [1], according to which the unexpected death of a loved one was the most frequently experienced traumatic event. Previously, South Koreans perceived unemployment or job loss and academic or work difficulties as traumatic events [18]. In the era of the Fourth Industrial Revolution, technological advances have caused changes in the number of jobs and duties in existing occupations globally, and these changes are also taking place in South Korea [27]. Therefore, the current results might be associated with employment difficulties and workplace competition in the context of the transforming employment market in South Korea. In fact, the most common traumas experienced by adults were different from the traumatic experiences that caused the most severe distress. Participants in this study reported death and disease in loved ones as the most distressing traumatic experiences in terms of perceived severity. Hence, healthcare providers should be informed about both kinds of adult traumatic experiences to enhance patient health management.

In this study, males reported more childhood trauma than females, including a higher prevalence of physical abuse, sexual abuse, and physical neglect. This evidence of differences in childhood trauma by gender is inconsistent with previous studies. While sexual, physical, and emotional abuse have been reported to be significantly higher in females [28], other studies have reported no significant differences in childhood trauma by gender [29,30]. The tendency to be abused as a child may vary depending on individual and cultural factors. It is known that boys are typically less obedient to their parents than are girls in preschool-age groups, which increases the likelihood of conflict and abuse in boys’ relationships with their parents as compared to that in girls’ relationships [31]. Further, there are cultural differences in forms of child discipline [31]. Thus, the higher level of childhood trauma in males observed in this study may have been influenced by various individual personalities and environmental and cultural factors during childhood. The current findings also indicate that the prevalence of childhood sexual abuse was higher in males than in females. However, this result is not consistent with the 2017 Korean national report of child abuse that found a greater proportion of sexual abuse cases among girls [32]. This result can be interpreted in two ways. First, the wording of sexual abuse items on childhood trauma measurement tools may affect sexual abuse scores. Questionnaires for measuring sexual abuse include questions such as “Someone tried to touch me in a sexual way or tried to make me touch them” and “Someone tried to make me do sexual things or watch sexual things.” These statements could refer to a variety of situations ranging in severity, but the findings suggest that males might experience milder sexually abusive behaviors than females during childhood. Second, if boys do experience more severe sexual abuse, they may be less likely to seek help than girls, leading to underreporting of such cases to the National Child Protection Agency. Further research is necessary to examine gender differences in childhood trauma experiences and their severity. 

The present findings indicate that females experienced more adulthood trauma-related distress than males did, in line with previous studies that reported a higher incidence of traumatic events in females [19,26], and the fact that females are more prone to developing PTSD [2,26]. Females are more likely to be victims of sexual abuse or domestic violence than males are, and they are also more likely to perceive and experience interpersonal relationship problems more intensely owing to their relationship-oriented tendencies [18]. Thus, the current results suggest that females may have an increased risk of and vulnerability to adulthood trauma. 

The current results showed that there were gender and age differences in trauma experiences. In the 19–29 and 30–39 age groups, males had significantly higher childhood trauma than females did, a possible explanation for which has been discussed above. Another noteworthy result was that the relation between age and childhood trauma was different according to gender. In males, higher childhood trauma was related to lower age. This suggests that the influence of recall bias could be lower in younger individuals than in older ones owing to differences in the perceived temporal distance of traumatic childhood memories [33]. In other words, younger males may have remembered their childhood trauma more clearly, making them more vulnerable to its negative effects. However, in females, higher childhood trauma was related to older age. This result is similar with a previous study that girls were more likely to present rising and sustained distress and boys were more likely to have declining distress [34]. Female trauma survivors tended to blame themselves more for their traumatic events, believed more that they were damaged, and recognized more that the world is dangerous than male survivors [35], and then such negative thoughts and emotions were remembered longer than neutral ones [36]. Considering this point of view, these results indicate that although females had a lower level of childhood trauma than males, it may last longer in older age. In addition, adulthood trauma was associated with being female or elderly across the study population. This could arguably be interpreted as an indicator of the mounting distress caused by the daily accumulation of traumatic events. Based on these findings, males, especially those aged 19–39 years, should be assessed for childhood trauma, and adulthood trauma should be considered in assessing older aged males. In females, both childhood trauma and adulthood trauma should be considered in assessing older aged females. Further research is needed to develop targeted preventive interventions to mitigate the negative effects of trauma and to promote physical and mental health among people with childhood or adulthood trauma.

The present study provided knowledge of the characteristics of traumatic experiences among South Korean adults. Specifically, this study investigated the frequencies of traumatic experiences and their differences by gender and age. There has been limited research about the various characteristics of traumatic experiences among the general South Korean population. Trauma-informed care provides services to individuals who were exposed to traumatic events based on knowledge and responsiveness of the role of trauma [3,15]. The efforts of addressing trauma needs to be provided in trauma-informed context of care, which is grounded on the understanding and knowledge of trauma to improve its effectiveness [3]. With a greater understanding of trauma, healthcare providers are becoming more trauma-informed [16,17]. Based on the current results of this study, understanding the frequency of traumatic experiences and differences according to gender and age may help improve awareness, assessment, and intervention through trauma-informed care in the public health system [15,16,17]. In addition, this study was based on a large community sample, compared to previous studies in South Korea. Thus, the current results reflected the diversity of people in South Korea.

Although these findings make an important contribution to the literature on traumatic experiences in South Korean adults, they should be interpreted within the context of certain limitations. First, as the questionnaire was administered on an online survey platform, it is likely that there was some sampling bias in that people with greater internet access were more likely to be recruited. Internet access may be insufficient or limited in low-economic or low-education groups, which may be related to trauma prevalence. Future studies should investigate the prevalence of trauma with representative community samples that are not restricted by Internet access in South Korea. Second, while we measured adulthood trauma experiences and distress levels, we did not measure the impact of the recurrence or frequency of each type of trauma, possibly underrepresenting the impact of repeated experiences of trauma. Further research is needed to develop an instrument measuring traumatic experiences to examine the interaction of frequency and distress level.

## 5. Conclusions

We examined the frequencies of traumatic experiences and their differences by gender and age in South Korean adults. Traumatic events were commonly experienced (79.5%). Unemployment or job loss was the most frequent type of adulthood trauma, while the unexpected death of loved ones was the most distressing. Childhood trauma was higher in males than in females; and in males, higher childhood trauma was related to younger age, conversely higher childhood trauma was related to older age in females. Adulthood trauma was associated particularly with females and being an older person. Trauma experiences should be further assessed in the context of gender and age to provide targeted interventions. The current findings can form the basis for improving the assessment of psychological trauma and providing targeted interventions through trauma-informed strategies in public health practice. 

## Figures and Tables

**Table 1:** Differences in trauma-related variables by sociodemographic characteristics (*N* = 3000).

Characteristics	*n* (%)	Childhood Trauma				Adulthood Trauma			
		Median (*IQR*)	Mean Rank	*U* or *χ*^2^*_kw_*(*df*)	*p*	Median (*IQR*)	Mean Rank	*U* or *χ*^2^*_kw_*(*df*)	*p*
Gender									
Male	1500 (50.0)	43 (33)	1547.59	1,054,371.50	0.003	9 (17)	1412.92	993,636.00	<0.001
Female	1500 (50.0)	42 (24)	1453.41			13 (19)	1588.08		
Age group (years)									
19–29	750 (25.0)	41 (28)	1468.19	9.37 (3)	0.025	9 (15)	1385.38	20.18 (3)	<0.001
30–39	750 (25.0)	44 (33)	1578.05			12 (18)	1499.58		
40–49	750 (25.0)	42 (27)	1503.29			12 (19)	1561.69		
50–65	750 (25.0)	41 (23)	1452.48			12 (19)	1555.34		
Living status									
With spouse	1596 (53.2)	41 (26)	1453.21	1,044,914.50	0.001	10 (17)	1439.23	1,022,598.00	<0.001
Without spouse	1404 (46.8)	44 (29)	1554.26			12 (19)	1570.15		
Education									
≤High school	515 (17.2)	47 (28)	1671.45	551,847.00	<0.001	13 (20)	1616.80	579,992.50	0.001
≥College	2485 (82.8)	41 (27)	1465.07			11 (18)	1476.40		
Employment status		00							
Unemployed	794 (26.5)	43 (27)	1519.49	860,706.50	0.471	11 (18)	1512.72	866,077.00	0.641
Employed	2206 (73.5)	42 (28)	1493.67			11 (17)	1496.10		
Economic status									
Low	898 (29.9)	47 (31)	1661.47	63.88 (2)	<0.001	13 (20)	1605.04	19.49 (2)	<0.001
Moderate	954 (31.8)	42 (26)	1523.44			10 (17)	1472.45		
High	1148 (38.3)	39 (25)	1355.51			10 (16)	1442.04		
**Total Median (*IQR*)**		42 (28)				11 (17)			
**Range**		25–111				0–70			

*IQR* = interquartile range; *df* = degrees of freedom; *p*-values were calculated using Mann–Whitney test or Kruskal–Wallis test.

**Table 2:** Characteristics of childhood and adulthood trauma and its differences by gender (*N* = 3000).

Categories	Total		Gender						
			Male (*n* = 1500)		Female (*n* = 1500)				
**Types of childhood trauma**	**Median (*IQR*)**		**Median (*IQR*)**	**Mean rank**	**Median (*IQR*)**	**Mean rank**	***U***	***p***	
Physical abuse	7 (7)		7 (8)	1580.25	6 (6)	1420.75	1,005,371.50	<0.001	
Emotional abuse	8 (7)		7 (8)	1472.06	8 (7)	1528.94	1,082,335.50	0.068	
Sexual abuse	5 (5)		5 (6)	1562.65	5 (3)	1438.35	1,031,776.00	<0.001	
Physical neglect	9 (7)		10 (6)	1619.57	9 (6)	1381.43	946,396.50	<0.001	
Emotional neglect	12 (7)		12 (7)	1497.50	12 (7)	1503.50	1,120,494.00	0.849	
**Types of adulthood trauma ^†^**	***n* (%)**		***n* (%)**		***n* (%)**		***χ*^2^ (*df*)**	***p***	**Distress (M ± *SD*) ^‡^**
Verbal threats or violence	789	(26.3)	381	(25.4)	408	(27.2)	1.25 (1)	0.263	3.77 ± 1.08
Physical threats or violence	437	(14.6)	236	(15.7)	201	(13.4)	3.28 (1)	0.070	3.74 ± 1.13
Victimization such as assault or robbery	142	(4.7)	80	(5.3)	62	(4.1)	2.39 (1)	0.122	3.82 ± 1.11
Sexual harassment or sexual abuse	290	(9.7)	63	(4.2)	227	(15.1)	102.67 (1)	<0.001	3.71 ± 1.24
Severe physical illness	422	(14.1)	224	(14.9)	198	(13.2)	1.86 (1)	0.172	3.97 ± 1.05
Disease in a loved one	736	(24.5)	368	(24.5)	368	(24.5)	0.00 (1)	0.998	4.32 ± 0.82
Unexpected death of a loved one	931	(31.1)	459	(30.6)	473	(31.5)	0.30 (1)	0.581	4.54 ± 0.76
Breakdown of an interpersonal relationship	1146	(38.2)	513	(34.2)	633	(42.2)	20.33 (1)	<0.001	3.96 ± 0.94
Bullying in school or at the workplace	465	(15.5)	185	(12.3)	280	(18.7)	22.96 (1)	<0.001	3.93 ± 0.98
Betrayal	807	(26.9)	370	(24.7)	437	(29.1)	7.60 (1)	0.006	4.15 ± 0.90
Divorce or separation (own or parents’)	401	(13.4)	156	(10.4)	245	(16.3)	22.80 (1)	<0.001	3.74 ± 1.20
Academic or work difficulties	1168	(38.9)	548	(36.5)	620	(41.3)	7.26 (1)	0.007	3.95 ± 0.90
Unemployment or job loss	1240	(41.3)	618	(41.2)	622	(41.5)	0.02 (1)	0.882	3.96 ± 0.91
Financial difficulties	895	(29.8)	441	(29.4)	454	(30.3)	0.26 (1)	0.604	4.30 ± 0.89
Witness or awareness of suicide of other persons	251	(8.4)	100	(6.7)	151	(10.1)	11.30 (1)	0.001	4.26 ± 0.86
Other traumatic events ^§^	3	(0.1)	1	(0.1)	2	(0.1)	0.33 (1)	0.564	5.00 ± 0.00
**Number of adulthood trauma experiences**									
At least 1 type of trauma	2386	(79.5)	1153	(76.9)	1233	(82.2)	13.10 (1)	<0.001	
None	614	(20.5)	347	(23.1)	267	(17.8)			
**Utilization of mental health services**								*n* (%)	
1. After any of the traumatic experiences mentioned above, did you use mental health services? (*n* = 2386) ^¶^									
No								2216	(92.9)
Yes								170	(7.1)
2. If you did not use mental health services, what was the reason? (*n* = 2216) ^||^									
Concerns about stigmatization or disadvantages in social life								148	(6.7)
Concerns about efficacy								193	(8.7)
Time or money constraints								240	(10.8)
Belief in healing with time								1425	(64.3)
Lack of information about services								210	(9.5)

*IQR* = interquartile range; *M* = mean; *SD* = standard deviation; *df* = degrees of freedom; *p*-values were calculated using Mann–Whitney test or *χ*^2^ tests; ^†^ Multiple answers allowed; ^‡^ *M* ± *SD* was based on the respondents who reported experiencing each type of adulthood trauma; ^§^ Other traumatic events included death of a pet, physical disability, and lawsuit; ^¶^ Respondents who reported at least one experience of adulthood trauma; ^||^ Respondents who did not use mental health services after traumatic experiences.

**Table 3:** Differences in childhood and adulthood trauma by gender and age.

Age	Male		Female			*p*
	Median (*IQR*)	Mean Rank	Median (*IQR*)	Mean Rank	*U*	
Childhood trauma						
19–29	43 (35)	396.87	41 (22)	354.13	62,298.50	0.007
30–39	49 (37)	400.01	43 (28)	350.99	61,121.50	0.002
40–49	42 (31)	386.45	42 (25)	364.55	66,205.50	0.166
50–65	41 (22)	364.46	42 (23)	386.54	66,171.50	0.163
Correlations between age and childhood trauma						
***r* (*p*)**	−0.07 (0.008)		0.05 (0.039)			
Adulthood trauma						
19–29	7 (15)	342.75	12 (16)	408.25	58,032.00	<0.001
30–39	9 (19)	347.60	14 (19)	403.40	59,850.50	<0.001
40–49	10 (20)	368.03	12 (20)	382.97	67,510.00	0.343
50–65	11 (19)	355.53	13 (19)	395.47	62,824.00	0.011
Correlations between age and adulthood trauma						
***r* (*p*)**	0.11 (< 0.001)		0.05 (0.036)			

*IQR* = interquartile range; *p*-values were calculated using Mann–Whitney test or Spearman’s rank correlation.
